# Seroprevalence and associated risk factors for HIV, Hepatitis B and C among blood Donors in South Gondar District blood Bank, Northwest Ethiopia

**DOI:** 10.1186/s12879-019-4051-y

**Published:** 2019-05-16

**Authors:** Markos Negash, Moges Ayalew, Demeke Geremew, Meseret Workineh

**Affiliations:** 1 College of Medicine and Health Sciences, School of Biomedical and Laboratory Sciences, University of Gondar, P O Box-196, Gondar, Northwest Ethiopia; 2 Debre Tabor Hospital, Debre Tabor, Northwest Ethiopia

**Keywords:** Blood transfusion, Transfusion-transmissible infection

## Abstract

**Background:**

Despite the undeniable significance of blood transfusion in saving a millions life in emergencies and medical treatment, the quality of blood faced challenges from transfusion-transmitted infections (TTIs) such as HIV (human immunodeficiency virus), HBV (hepatitis B virus) and HCV (hepatitis C virus). This cross-sectional study was undertaken with the aim of determining the seroprevalence and risk factors of HIV, HBV, and HCV among blood donors.

**Methods:**

An institutional based cross-sectional study was conducted at Debre Tabor district hospital from January 2017 to February 2018. Blood samples from volunteer donors collected; serum separated and screened with ELISA tests for detection of anti-HIV, hepatitis-B surface antigen (HBsAg) and anti-HCV. Fishers’ exact test was employed to see the association between variables as well logistic regression tests were applied to identify potential risk factors. *P*-value of less than 0.05 was considered as statistically significant.

**Result:**

A total of 310 volunteer donors were included in the study. The proportion of blood donors having at least one viral-TTI was 12.6% while the magnitudes of HIV, HBV, and HCV were 2.6, 5.8 and 4.2%, respectively. Educational status and multiple sexual behaviors are significantly associated with HIV acquisition whilst marital status was significantly associated with HBsAg seropositivity.

**Conclusion:**

Seroprevalence of transfusion-transmissible infections was high and alarming therefore proper screening of donated blood with test methods having better diagnostic performance should be employed. Also encouragement of blood donation from voluntary donors and creating awareness on the general public regarding HIV, HBsAg and HCV transmission and prevention should be strengthen.

## Background

Blood transfusion is a life-saving therapeutic intervention in which millions of lives are saved each year globally [1]. However, access to safe blood and blood products still remains a major challenge throughout the world, especially in developing countries. Blood can be a vector for harmful transfusion-transmissible infections (TTIs) and numerous viral pathogens affects the safety of blood intended for transfusion these includes Human immunodeficiency virus (HIV), hepatitis B virus (HBV), and hepatitis C virus (HCV) [2–4].

The World Health Organization (WHO), to assure the quality and safety, recommends screening of donated blood for a minimum of the major TTIs [5]. Proper donor selection and examination of the donated blood for TTIs evidently reduced the transmission of these infections in the past 20 years [6, 7]. Nevertheless, the safety of blood for transfusion in developing country is still questionable due to the growing magnitude of viral infections (HIV, HBV and HCV) in the population, as well as screening with poor quality of screening tools [8, 9].

Viral TTIs varies globally, in Africa alone, up to 500 peoples acquire TTIs due to contaminated blood transfusion daily [10]. Sub-Saharan Africa inhabited with 38 million HIV infected peoples, remains one of the highest regions as 68% of globally HIV infected people are residing in this region consequently resulting about 15% of HIV transmission through blood transfusion [11, 12]. Global distribution of HBV and HCV infection among blood donors is variable. Findings from Asian study indicated that the magnitude of HBV HCV among blood donors were 1.76 and 0.19% [13], while 0.51 and 0.25% of blood donors in China were positive for HBsAg and HCV respectively [14].

The demand for blood transfusion in Ethiopia has been reported to be high as a result of frequent road traffic accidents, surgical and obstetric blood loss, as well as anemia from other sources [15–17]. Epidemiological studies conducted on TTIs in several regions of Ethiopia showed a magnitude of 43.2, 11.5, 7.0, 5.9, and 3.6% in Bahir Dar, Jijiga, Hawassa, north Gondar, and Jimma respectively [18–22]. Currently, in Ethiopia, several programs are designed and implemented to reduce the transmission and incidence of HBV that includes early child hood vaccination, screening of all pregnant women for HBsAg, and vaccination of high risk target groups like healthcare providers. However, according to WHO, Ethiopia is categorized under regions where viral hepatitis infection ranging from intermediate to hyper-endemic [23]. A systematic review and meta-analysis study conducted in Ethiopia revealed that HBV is highly prevalent among blood donors (8.4%) next to immigrants (11.0%) [24]. It is also documented that viral hepatitis is the most predominant viral-TTI than HIV and HCV among blood donors [18–22].

Assessment of viral-TTIs and potential risk factors is the mainstay for providing a clear picture on the prevalence of these infections among blood donors which ultimately evaluate preventive strategies and take corrective measures. Consequently, data obtained from the study will permit to see the safety of donated bloods for transfusion there by health policy makers may consider a better diagnostic scheme. Also, evaluation of potential risk groups and the extent of factors will strengthen the decision on selection of low-risk donor, proper collection and application of better screening methods thereby, safe and adequate blood donation for transfusion will be guaranteed. However, data are hardly limited in South Gondar, Northwest Ethiopia. Therefore, the objective of the current study was to determine the seroprevalence HIV, HBV, and HCV as well as potential risk factors among blood donors at South Gondar district Blood Bank, northwest Ethiopia.

## Methods

### Study design and setting

A cross-sectional study was conducted at Debre Tabor Hospital blood bank from January 2017 to February 2018. The hospital is a tertiary level hospital that provides health service to over three million inhabitants and residents found in Debre Tabor city, South Gondar Administrative Zone, Amhara National Regional State, 666 Km north from the capital city, Addis Ababa. The blood bank was established in 2014 G.C.

### Study subjects and sampling

For this study, based on the criteria established by the Ethiopian Red Cross Society, all voluntary blood donors who were eligible and donated blood during the study period were included. As the new WHO guide line encourages voluntary blood donation, consecutive blood donors from January 2017 to February 2018 were incorporated.

### Sample collection and laboratory analysis

Aliquots of whole blood were taken from blood donors and serological assays for HIV infection [3rd generation ELISA, Vironostika HIV Uni-Form II AG (Bio-Merieux, Boxtel, Netherlands)], HBV [ELISA Hepanostika HBsAg UNi-Form II (Bio-Merieux, Boxtel, Netherlands)] and HCV [Human anti-HCV 3rd generation ELISA (Human Gasellschaft for Bio-chemical and diagnostic MbH, Germany)] were screened at Debre Tabor hospital blood bank. All the blood screening, sample processing and interpretation of test result is performed according to the manufacturer’s instruction. Additionally, data regarding socio-demographic and potential risk factors for HIV, HBV and HCV were collected from each volunteer blood donors using a pre-tested structured questionnaire (questioners in this study, in addition to the routinely used, are modified and designed in a way that assuring collection of relevant associated factors for this study purpose) at the time of blood collection.

### Quality control and data analysis

We strictly followed standard operational procedures during sample collection and laboratory investigation in order to maintain the quality of the study. Known positive and negative samples for HIV, HBV and HCV were used as an internal quality control; in addition, all laboratory analysis was performed according to the manufacturers’ instruction. The data were double checked, entered and analyzed using SPSS version 20.0 statistical software. Fishers’ exact test, logistic regression and 95% confidence interval were employed for group comparison of blood donors and association of variables. Also, *p* value of less than 0.05 was considered as statistically significant.

## Result

### Demographic characteristics

A total of 338 voluntary blood donors, as non-remunerated donor found, were identified as blood donors. However, 28 participants were not eligible for blood donation (based on the criteria set by Ethiopian Red Blood Society) hence blood sample from 310donors were screened for HIV, HBV and HCV at Debre Tabor blood bank from September 2016 to July 2017. Among them 198(63.8%) were males and 112(36.2%) were females. The mean ± SD age of the study participants was 23.1 ± 3.02 and 69.0% of the blood donors were from urban areas (Table 1).

**Table 1:** Soci-odemographic status of blood donors at Debretabor General Hospital, South Gondar, Northwest Ethiopia, 2017. N, 310

Variables		Frequency	Percentage (%)
Gender	Male	198	63.8
	Female	112	36.2
Age (years)	18–27	128	41.4
	28–37	112	36.1
	38–47	55	17.6
	> 48	15	4.9
Resident	Urban	214	69.0
	Rural	96	31.0
Occupation	Employed	143	46.1
	Day laborer	19	6.1
	House wife	10	3.3
	Student	132	42.4
	Others	6	2.1
Religion	Orthodox	242	78.0
	Muslim	52	16.7
	Catholic	10	3.3
	Others	6	2.0

### Viral TTIs and associated risk factors among voluntary blood donors

The proportion of voluntary blood donors with at least one TTI marker was 12.6% while the magnitudes of HIV, HBV and HCV among blood donors were 2.6, 5.8 and 4.2%, respectively. Seropositivity for either of HIV, HBV and HCV is more common in males than females; HIV is more prevalent in 18–27 years younger age groups, whereas both HBV and HCV are prevalent in 28–37 years older age groups (Table 2).

**Table 2:** Prevalence of TTIs in different soci-odemographic variables among blood donors at Debretabor General Hospital, South Gondar, Northwest Ethiopia, 2017. N, 310

Variables		HIV positive N (%)	HBV Positive N (%)	HCV Positive N (%)
Gender	Male	6(3.03)	12(6.06)	9 (4.54)
	Female	2 (1.78)	6(5.35)	4 (3.57)
Age (years)	18–27	5(3.90)	6 (4.68)	3 (2.34)
	28–37	1 (0.78)	8 (7.14)	7 (6.25)
	38–47	1 (1.81)	3 (5.45)	2 (3.63)
	> 48	1(6.66)	1 (6.66)	1 (6.66)
Resident	Urban	6 (2.80)	13 (6.07)	10 (4.67)
	Rural	2 (2.08)	5 (5.20)	3 (3.12)
Occupation	Employed	4 (2.06)	8 (5.60)	4 (2.80)
	Day laborer	3(7.69)	5 (20.0)	5 (20.0)
	House wife	–	1 (10.0)	1 (10.0)
	Student	1(1.12)	4 (3.0)	3 (2.20)
Religion	Orthodox	7 (2.89)	13 (5.37)	10 (4.13)
	Muslim	1 (1.92)	4 (7.69)	2 (3.84)
	Catholic & others	–	1 (6.25)	1 (6.25)

With respect to the plausible risk factors, the bivariate analysis showed that occupation, history of invasive procedure, history of tattooing, and needle-stick injuries were insignificantly associated with HIV infection. However, multivariate analysis showed that being illiterate and history of having multiple sexual partners was significantly associated with HIV sero-positivity (Table 3).

**Table 3:** Prevalence and multiple logistic regression observations on risk factors associated with HIV infection among blood donors at Debretabor General Hospital, South Gondar, Northwest Ethiopia, 2017, N, 310

Variables		Total	Anti-HIV status	COR (95%CI)	*P*-value	AOR (95%CI)	*P*-value
			Positive (%)				
Occupation	Employed	143	4 (2.8)				
	Day laborer	25	3 (12.0)				
	House wife	10	0				
	Student	132	1 (0.8)				
Educational status	Illiterate	25	2 (8.0)	1.70 (1.02–2.78)	0.04	1.20(0.40–5.61)	0.04
	Primary school	85	1 (1.2)	12.1(2.30–25.7)	0.10	15.4(4.30–129)	0.07
	Secondary and above	200	5 (2.5)	1			
Marital status	Single	175	4 (2.3)	1			
	Married	96	1 (1.0)	2.10(1.08–3.39)	0.63		
	Widowed	29	2 (6.9)	4.91(2.11–7.03)	0.42		
	Divorced	10	1 (10.0)	1.80(0.45–3.99)	0.07		
Previous history of BT	Yes	60	1 (1.7)	1.34(0.59–2.33)	0.41		
	No	250	7 (2.8)	1			
Multiple sexual partner	Yes	81	7 (8.7)	2.44(1.19–4.26)	0.02	1.82(0.91–25.1)	0.04
	No	229	1 (0.5)	1			
History of invasive procedure	Yes	12	0				
	No	298	8 (2.7)				
History of tattooing	Yes	34	2 (5.9)	1.22(0.52–2.79)	0.55		
	No	276	6 (2.2)				
Shaving habit	Yes	49	2 (4.1)	0.73(0.21–1.77)	0.65		
	No	261	6 (2.3)				
Needle-stick injury	Yes	97	3 (3.1)	2.11(1.39–3.34)	0.85		
	No	213	5 (3.3)	1			
Communal use of sharp materials	Yes	123	1 (0.8)	1.01(0.30–2.16)	0.95		
	No	187	7 (3.7)				
Sharing of toothbrush	Yes	67	2 (3.0)	1.19(0.72–2.17)	0.50		
	No	243	6 (2.5)	1			
Ear pricing	Yes	87	1(1.1)	2.10(0.57–6.87)	0.23		
	No	223	7 (3.1)	1			

*HIV* human immunodeficiency virus, *COR* crude odds ratio, *AOR* adjusted odds ratio, *CI* confidence interval

Regarding on the sero-positivity of HBsAg, neither of having multiple sexual partner, tattooing, needle-stick injury, and ear pricings were significantly associated with HBV infection. But, marital status (being unmarried/single) is significantly associated with HBsAg. The odds of study participants with the history of marital status being single (AOR = 2.01, 95% *CI* = 0.97–3.78, *P* = 0.023) is around two times higher for of acquiring HBV infection that those who were married or couple (Table 4). Higher magnitude of anti-HCV was seen among blood donors next to HBsAg sero-positivity, but all analyzed variables did not show any statistically significant association with respect to seropositivity for HCV. Despite that no risk factor is significantly associated, higher magnitude of HCV sero-positivity was seen among day laborers and donors with multiple sexual partners (Table 5).

**Table 4:** Prevalence and multiple logistic regression observations on risk factors associated with Hepatitis B surface antigen infection among blood donors at Debretabor General Hospital, South Gondar, Northwest Ethiopia, 2017, N, 310

Variables		Total	HBsAg status	COR (95%CI)	*P*-value	AOR (95%CI)	*P*-value
			Positive (%)				
Occupation	Employed	143	8 (5.6)	1.72(0.61–6.96)	0.35		
	Day laborer	25	5 (20.0)	1.20(0.42–4.50)	0.69		
	House wife	10	1 (10.0)	3.11(0.30–13)	0.15		
	Student	132	4 (3.0)	1			
Educational status	Illiterate	25	2 (8.0)	1.73(0.61–6.75)	0.35		
	Primary school	85	4 (4.8)	3.70(1.20–9.03)	0.45		
	Secondary and above	200	12 (6.0)	1			
Marital status	Single	175	7 (4.0)	2.12(1.42–4.59)	0.01	2.01(0.97–3.78)	0.02
	Married	96	4 (6.2)	1			
	Widowed	29	4 (13.8)	3.16(0.56–12.4)	0.19	2.45(0.40–5.96)	0.09
	Divorced	10	1 (10.0)	0.21-(0.30–1.0)	0.17	1.40(0.59–4.57)	0.79
Previous history of BT	Yes	60	2 (3.3)	1.67(1.25–3.92)	0.63		
	No	250	16 (6.4)	1			
Multiple sexual partner	Yes	81	11 (10.0)	1.02(0.57–2.6)	0.87		
	No	229	7 (3.5)	1			
History of invasive procedure	Yes	12	1 (8.3)	1.59(0.79–2.99)	0.11	1.25(0.40–5.20)	0.50
	No	298	17 (5.7)	1			
History of tattooing	Yes	34	3 (8.8)	3.56(1.47–6.19)	0.45		
	No	276	15 (5.4)	1			
Contact with family having liver disease	Yes	49	2 (4.0)	1.48(0.88–2.57)	0.07	1.02-(0.01–2.07)	0.60
	No	261	16 (6.1)	1			
Needle-stick injury	Yes	97	4 (4.1)	1.18(0.68–4.49)	0.68		
	No	213	14 (6.6)	1			
Communal use of sharp materials	Yes	123	3 (2.4)	3.11(0.79–13.7)	0.14		
	No	187	15 (8.0)	1			
Ear pricing	Yes	87	2 (2.3)	1.68(0.73–2.51)	0.24		
	No	223	16 (7.2)	1			

*HBsAg* hepatitis B surface antigen, *COR* crude odds ratio, *AOR* adjusted odds ratio, *CI* confidence interval

**Table 5:** Prevalence and multiple logistic regression observations on risk factors associated with HCV among blood donors at Debretabor General Hospital, South Gondar, Northwest Ethiopia, 2017, N, 310

Variables		Total	Anti-HCV status	COR (95%CI)	*P*-value	AOR (95%CI)	*P*-value
			Positive (%)				
Occupation	Employed	143	4 (2.8)	0.48 (0.15–3.56)	0.47		
	Day laborer	25	5 (20.0)	0.70(0.05–5.90)	0.87		
	House wife	10	1 (10.0)	2.70(0.93–5.09)	0.99		
	Student	132	3 (2.2)	1			
Educational status	Illiterate	25	1 (4.0)	0.77 (0.18–7.40)	0.87		
	Primary school	85	4 (4.7)	3.81(0.9–8.60)	1.97		
	Secondary and above	200	8 (3.5)	1			
Marital status	Single	175	6 (3.4)	2.74 (0.61–4.76)	0.08	1.73(0.27–4.90)	0.17
	Married	96	3 (3.1)	6.74(1.90–21.9)	0.04	0.26(0.15–2.96)	0.95
	Widowed	29	2 (6.9)	1.81(.68–4.37)	0.18	0.39(0.11–0.97)	0.55
	Divorced	10	1 (10.0)	1			
Previous history of BT	Yes	60	1 (1.7)	1.50 (0.40–4.91)	0.77		
	No	250	12 (4.8)	1			
Multiple sexual partner	Yes	81	9 (11.1)	1.32 (0.37–3.96)	0.10		
	No	229	4 (1.7)	1			
History of invasive procedure	Yes	12	`0				
	No	298	13 (4.4)				
Contact with family having liver disease	Yes	49	3 (8.2)	2.22(1.34–4.89)	0.29		
	No	261	10 (3.8)	1			
Needle-stick injury	Yes	97	1 (1.0)	1.62(0.45–3.73)	0.56		
	No	213	12 (5.6)	1			
Communal use of sharp materials	Yes	123	2 (1.6)	3.77(1.29–10.21)	0.77		
	No	187	11 (5.9)	1			
Ear pricing	Yes	87	1 (1.5)	2.44(1.23–4.80)	0.55		
	No	223	12 (5.4)	1			

*HCV* hepatitis C virus, *COR* crude odds ratio, *AOR* adjusted odds ratio, *CI* confidence interval

## Discussion

Developing countries, like Ethiopia, are constantly facing insufficient supply of safe blood for transfusion and an increase in the magnitude of TTIs. The WHO encourages blood donation from voluntary donors because this kind of donation believed to have lesser chance harboring and transmitting TTIs [25]. The donor distribution in this study showed a predominantly blood donors aged from 18 to 27 in our donor pool. Studies ( [21, 22], and [26]) had reported a similar age distribution among blood donors in Ethiopia. This similarity in age distribution is due to the fact that as it represents the most active age stratum of the population and participates vigorously in blood donation.

This study demonstrated that the overall sero-prevalence of blood-borne viral infections (HIV, HBV and HCV) in the donor population is 12.6%. This finding is somewhat similar with that of studies conducted in Ethiopia and other developing countries [19, 27–29] whereas we found a lower prevalence of TTIs as compared with domestic studies conducted in Ethiopia (29.5 and 43.2%) and other African countries (19.3, 26.2, 28.8%) respectively [18, 30–33]. This is probably because of the actual differences in study population, design, duration, even occupational variation and geographical differences of the study populations; moreover, the prescreening procedure may also play a role for the observed variations. This study further focused on volunteer donors that might decrease the prevalence rates of TTIs from other studies. Additionally, the lower prevalence of TTIs in this study may be due to the fact that this study is focused only on three viral infections (i.e. HIV, HBV, and HCV), while other studies incorporated magnitude of syphilis in addition to viral infections.

In this study, known that there is a similarity between risk factors and route of transmission between HIV, HBV and HCV, we found that the prevalence of HBV was higher as compared to HCV and HIV. Several studies also come up with similar findings compared with the present study [1, 21, 34–37]. The probable reason for this high prevalence may be due to higher infectivity of HBV compared to HCV and HIV as well as poor awareness of the community towards hepatitis transmission and infection. Although there is a recent advent of Hepatits B vaccination in the country, vaccination is freely accessible for only health professionals while the rest of community members have to pay to get this vaccination. This and the mis-perception towards hepatitis vaccination protective effect may play a significant role for the observed elevated HBV magnitude.

The prevalence of HIV in this study is 2.6% which is lower as compared with 3.8% from Northwest Ethiopia [26], 5.1% Dessie [38], 11.7% Bahir Dar [18] as well as several African studies including 3% reported from Sudan [39], 3.8% in Ghana [40], and 10.6% in Nigeria [41], also lower than 5.5% observed in Maiduguri [42], and 5.3% showed by study conducted at neighbor Kenya [43]. This lower prevalence is may be due to the reason that the above studies were performed on the period where HIV magnitude reached peak and variation in the sensitivity and specificity of screening tools applied in blood bank centers/general population of those countries as well as diagnostic algorithms used in each study. It should be noted that screening algorithms of HIV is frequently changing and modified in several African countries, including Ethiopia, resulting a variable diagnostic performance of test kits that ultimately provide characteristically variable magnitude between countries.

Despite the 2.6% HIV sero-positivity in our study which is lower than from several sub-Saharan Africa countries, where HIV prevalence ranges from 3 to 5% [44], it is much higher than 0.1% Jijiga Ethiopian Somali region [19], a 1.6% magnitude from both Yrgalem and Hawassa studies, South Ethiopia [20, 45] 0.18, 1 and 1.13% reports from Eritrea, Nyala Hospital western Sudan and South Africa respectively [34, 35, 46], 0.4% in Khartoum [47], and from Egypt in which it was no cases reported [48]. The higher report from our study is probably due to the rise in seropositivity of HIV among the general population recently, the highest geographic distributions of HIV infection in the study area which is similar to Ethiopian national data [49]. As well variation in the burden of the disease in the society, population differences regarding social behavior, lifestyle, socioeconomic status, level of awareness and variation in study setting plus a stringent nonstop creation of awareness on HIV in the previously stated study might also explain in-part for the observed variations.

Hepatitis B is one of the most infectious diseases; reaching over 2 billion infected cases worldwide but it is hyperendemic in sub-Saharan Africa and Asia [34] and transmission of hepatitis B or hepatitis C due to contaminated blood is estimated to reach more than 45,000 in Saharan Africa annually [50]. The highest prevalence rates of TTIs among our donors were observed for hepatitis B and C infections than HIV (5.8 and 4.2%, respectively Table 2). This observations, somewhat, agrees with a prevalence of HBV in the Ethiopian general population which was reported to be between 6.1 and 7.4% [51, 52] and similar reports in Ethiopian blood donors as well as other African studies [30, 53, 54]. But our study demonstrated a lower prevalence in comparisons with a study conducted at Bahir Dar 25% [18], Jijga (10. 9%) [19], Wolaita sodo 9.5% [30], Bale Zone, South East Ethiopia (7.4%) [55], southern Sudan study which was 26% [56] 10% reported at Sudan [57], and Nigerian study [58, 59]. On the other hand our study revealed the highest magnitude of HBV among blood donors than previously reported studies in Ethiopia; Jimma (3.05%), North Gondar (3.6 and 4.7%), Southern Ethiopia (4.2 and 4.8%) [20–22, 26, 45] as well, in Eritrea 4% [34] northern and central Sudan, 5.1 and 5.6% respectively [60, 61]. The high prevalence of HBV as compared with the previous studies might be due to taking into consideration that HBV is highly infectious and transmission is getting elevated recently, variability in study settings and study period as our study used data collected in 2017 which is by far recent than the above studies.

Global estimation of Hepatitis C Virus (HCV) infection is thought to be over 170 million [62]. In this study 13/310 (4.2%) donors were confirmed positive for HCV infection which is somewhat similar to reports from Southeast Ethiopia 3.6% [63], Adwa 4.3% [64], 3.4 and 4.8% at Sudan and Cameroon respectively [65, 66]. But the result is by far higher than studies conducted in Amhara and Tigray regions 1.7% [17], eastern Ethiopia Jijiga 0.4% [67] and elsewhere 1.1% [68], 0.51% [69], 0.48% [70], 0.21% [71], 0.2% [72], 0.16% [73], and 0.03% [74]. The higher prevalence of this study as compared with the above findings could be supported by the reason that most of the studies were performed earlier at which magnitude of HCV is quite insignificant as well our finding revealed that despite the lower prevalence as compared to HBV, HCV infection is showing an increment in incident among blood donors.

There has been little documentation especially in the study area regarding on the risk factors of viral TTIs among blood donors. This study explains the occurrence of possible risk factors reported by blood donors with positive anti-HIV, HBV and HCV results (Tables 3, 4 and 5). Factors significantly associated with HIV infection were educational status and history of multiple sexual behaviors (*p < 0.045* and *p < 0.047* respectively). Conversely, no association was observed between HIV sero-positivity and history of operation, tattooing, shaving habit, needle injury, and other factors. Regarding the donors profile with respect to HBV, sero-positivity for HBsAg was significantly associated with marital status (OR 2.01; *p* < 0.023) by which many of the infected donors were among unmarried (single) group. These important findings will provide information by which screening of blood donated for transfusion should utilize the most advanced technique and high-performance kits with an emphasis on each donor group.

## Conclusion

The magnitude of transfusion-transmissible viral infections in blood donated at South Gondar district blood bank is high, known that all donors are voluntary, therefore proper screening of donated blood for HIV, HBsAg and HCV with test methods having better diagnostic performance should be employed which also decreases the rate of undetectable transmission (either due to window period or poor detection of the test) of viral infections. Stringent donor selection, encouragement of blood donation from voluntary donors and creating awareness on the general public regarding HIV, HBsAg and HCV transmission and prevention should be strengthened.

## Limitation of the study

This study did not observe the magnitude of TTIs other than HIV, HBV and HCV like syphilis and other infections. Also, the study period and participants are too small that makes generalization based on the observed result very difficult.

## abbreviations

Ab: Antibody
Ag: Antigen
HbsAg: Hepatitis-B surface antigen
HBV: Hepatitis B virus
HCV: Hepatitis C virus
HIV: Human immunodeficiency virus
TTIs: Transfusion-transmissible infections
WHO: Health Organization

## References

[1] Arora D, Arora B, Khetarpal A (2010). Seroprevalence of HIV, HBV, HCV and syphilis in blood donors in southern Haryana. Indian J Pathol Microbiol.

[2] Amiwero CE, Prescott RJ, George OA, Joy NI, Aisha M (2013). Seroprevalence of transfusion transmissible infections among blood donors attending the federal medical Centre. Bida IJMBR.

[3] Song Y, Bian Y, Petzold M, Ung COL (2014). Prevalence and trend of major transfusion transmissible infections among blood donors in Western China, 2005 through 2010. PLoS One.

[4] Rerambiah LK, Rerambiah LE, Bengone C, Siawaya JD (2014). The risk of transfusion transmitted viral infections at the Gabonese National Blood Transfusion Centre. Blood Transfus.

[5] Deshpande RH, Bhosale S, Gadgil P, Sonawane M (2012). Blood donor’s status of HIV, HBV, HCV and syphilis in this region of Marathwada, India. JKIMSU.

[6] Moiz B, Ali B, Chatha MH, Raheem A, Zaheer HA (2015). HIV prevalence in blood donors and recipients in Pakistan: a meta-analysis and analysis of blood-bank data. WHO South East Asia J Public Health.

[7] Mavenyengwa RT, Mukesi M, Chipare I, Shoombe E (2014). Prevalence of human immunodeficiency virus, syphilis, hepatitis B and C in blood donations in Namibia. BMC Public Health.

[8] Field SP, Allain JP (2007). Transfusion in sub-Saharan Africa: does a Western model fit?. J Clin Pathol.

[9] Lathamani K, Bhaktha G, Nayak S, Kotigadde S (2013). Prevalence of HIV, HCV, HBV and syphilis in blood donors among the Dakshina Kannada District, India. Int J Curr Microbiol App Sci.

[10] Walana W, Ahiaba S, Hokey P, Vicar EK, Acquah SEK, Der EM (2014). Sero-prevalence of HIV, HBV and HCV among blood donors in the Kintampo municipal hospital, Ghana. Brit Microbiol Res J.

[11] Fleming AF (1997). HIV and blood transfusion in sub-Saharan Africa. Transfus Sci.

[12] UNAIDS (2002). Report on the global AIDS epidemic.

[13] Kumar A, Shatish MS, Narayan SI, Nitin G (2014). Seroprevalence of transfusion transmissible infections (TTIs) among blood donors in a tertiary care hospital, Central India: a prospective study. Muller J Med Sci Res.

[14] Zheng X, Ding W, Li G (2015). Seroprevalence of transfusion-transmissible infectious agents among volunteer blood donors between 2006 and 2012 in Zhejiang, China. Blood Transfus.

[15] Ghendon Y (1987). Perinatal transmission of hepatitis B virus in high incidence countries. J Virol Methods.

[16] WHO. Global surveillance and control of hepatitis C. Report of a WHO consultanion organized in collaboration with the viral hepatitis prevention board, Antwerp, Belgium. J Viral Hepat. 6:35–47. 10.1046/j.1365-2893.1999.6120139.x PubMed: 10847128. 10847128

[17] Babatunde OM, Adedayo OF, Usen AU, Babatunde AO, Isreal E, Joseph IO (2015). Seroprevalence of transfusion transmissible infections (TTI), in first time blood donors in Abeokuta, Nigeria. Afr Health Sci.

[18] Dessie A, Abera B, Wale F (2007). Seroprevalence of major blood-borne infections among blood donors at Felege Hiwot referral hospital, Northwest Ethiopia. Ethiop J Health Dev.

[19] Mohammed Y, Bekele A (2016). Seroprevalence of transfusion transmitted infection among blood donors at Jijiga blood bank, eastern Ethiopia: retrospective 4 years study. BMC Res Notes.

[20] Birhaneselassie M (2016). Prevalence of transfusion-transmissible infections in donors to an Ethiopian blood Bank between 2009 and 2013 and donation factors that would improve the safety of the blood supply in underdeveloped countries. Lab Medicine.

[21] Belete B, Elias S, Berhanu W, Kefyalew A, Mulugeta M (2017). Transfusion-transmissible viral infections among blood donors at the North Gondar district blood bank, Northwest Ethiopia: a three year retrospective study. Transfusion-transmissible viral infections among blood donors at the North Gondar district blood bank, Northwest Ethiopia: a three year retrospective study. PLoS One.

[22] Wakjira K, Zeleke M, Asfaw G, Gemeda A (2017). Transfusion-transmissible infection surveillance among blood donors in Southwest Ethiopia: A six years retrospective study. Asian Pac J Trop Dis.

[23] WHO. Global policy report on the prevention and control of viral hepatitis in WHO Member States. Available at: http://www.who.int/hiv/pub/hepatitis/global_report/en/. Accessed on: 7/31/2018.

[24] Belyhun Y, Maier M, Mulu A, Diro E, Liebert UG (2016). Hepatitis viruses in Ethiopia: a systematic review and meta-analysis. BMC Infect Dis.

[25] WHO. Universal Access to Safe Blood; 2010. Available from: http://who.int/bloodsafety/universalbts/en/index.html. [Last accessed on 2018 Jan 10].

[26] Belay T, Gizachew Y, Afework K, Anteneh A, Andargachew M, Frank E, Ulrich S (2010). Seroprevalence of HIV, HBV, HCV and syphilis infections among blood donors at Gondar University Teaching Hospital, Northwest Ethiopia: declining trends over a period of five years. BMC Infect Dis.

[27] Matee MI, Magesa PM, Lyamuya EF (2006). Seroprevalence of human immunodeficiency virus, hepatitis B and C viruses and syphilis infections among blood donors at the Muhimbili National Hospital in dare Es Salam, Tanzania. BMC Public Health.

[28] Jocelijn S, Philippe G, Anja DW, Esther CC, Rosa M, Adelino JB (2011). Seroprevalence of transfusion-transmissible infections and evaluation of the pre-donation screening performance at the provincial hospital of Tete, Mozambique. BMC Infect Diseases.

[29] Usman W, Haroon K, Humayoon SS, Muhammad AA, Muhammad AM, Hasan AZ (2012). Prevalence of transfusion transmitted infections among blood donors of a teaching Hospital in Islamabad. Ann Pak Inst Med Sci.

[30] Bisetegen FS, Bekele FB, Ageru TA, Wada FW (2016). Transfusion-transmissible infections among voluntary blood donors at Wolaita Sodo University teaching referral hospital, South Ethiopia. Can J Infect Dis Med Microbiol.

[31] Nwankwo E, Mamodu I, Umar I, Musa B, Adeleke S (2012). Seroprevalence of major blood-borne infections among blood donors in Kano, Nigeria. Turk J Med Sci.

[32] Noubiap JJ, Joko WYA, Nansseu JRN, Tene UG, Siaka C (2013). Sero-epidemiology of human immunodeficiency virus, hepatitis B and C viruses, and syphilis infections among first time blood donors in EdeÂa, Cameroon. Int J Infect Dis.

[33] Makroo RN, Hegde V, Chowdhry M, Bhatia A, Rosamma NL (2015). Seroprevalence of infectious markers & their trends in blood donors in a hospital based blood bank in North India. Indian J Med Res.

[34] Fessehaye N, Naik D, Fessehaye T (2011). Transfusion transmitted infections—a retrospective analysis from the National Blood Transfusion Service in Eritrea. Pan Afr Med J.

[35] Abu M, Eltahir Y, Ali A (2009). Seroprevalence of hepatitis B virus and hepatitis C virus among blood donors in Nyala, South Darfur, Sudan. Virol J.

[36] Adekeye AM, Chukwuedo AA, Zhakom PM, Yakubu RS (2013). Prevalence of hepatitis B and C among blood donors in Jos south LGA, plateau state, Nigeria. Asian J Med Sci.

[37] Fatemeh F, Reza T, Saeed T, Marziyeh G, Gholamreza H, Nasrin S, Sakineh T, Abdolreza N (2016). Prevalence and trends of transfusion-transmissible viral infections among blood donors in south of Iran: an eleven-year retrospective study. PLoS One.

[38] Sharew B, Mulu A, Teka B, Tesfaye T (2017). HIV-Sero- prevalence trend among blood donors in north East Ethiopia. Afri Health Sci.

[39] Abdallah TM, Ali AAA (2012). Sero-prevalence of transfusion-transmissible infectious diseases among blood donors in Kassala, eastern Sudan. J Med Med Sci.

[40] Ampofo W, Nii-Trebi N, Ansah J, Abe K, Naito H, Aidoo S, Nuvor V, Brandful J, Yamamoto N, Ofori-Adjei D, Ishikawa K (2002). Prevalence of blood borne infectious diseases in blood donors in Ghana. J Clin Microbiol.

[41] Amadi AN, Mba LE (2001). Distribution of HIV infection in Abia state, Nigeria. Niger J Med Invest Pract.

[42] Baba MM, Hassan AW, Gashau W (2000). Prevalence of hepatitis B antigenaemia and human immunodeficiency virus in blood donors in Maiduguri, Nigeria. Niger J Med.

[43] Kamande MW, Kibebe H, Mokua J (2016). Prevalence of transfusion transmissible infections among blood donated at Nyeri satellite transfusion Centre in Kenya. IOSR J Pharm.

[44] Bloch EM, Vermeulen M, Murphy E (2012). Blood transfusion safety in Africa: a literature review of infectious disease and organizational challenges. Transfus Med Rev.

[45] Fisseha B, Mintewab H, Jemal A, Daniel G, Misganaw B (2017). The prevalence of transfusion transmitted infections: a focus on hepatitis B virus among blood donors at Hawassa blood bank center, southern Ethiopia. Int J Blood Transfus Immunohematol.

[46] Marion V, Ronel S, Dhuly C, Charlotte I (2017). Use of blood donor screening to monitor prevalence of HIV and hepatitis B and C viruses, South Africa. Emerg Infect Dis.

[47] Nmariq OA, Eiman DO, Musa AA (2016). Sero-prevalence of transfusion transmissible infections among blood donors in Khartoum Central Sudan. Eur Acad Res.

[48] Nada HA, Atwa M (2013). Seroprevalence of HBV, HCV, HIV and syphilis markers among blood donors at Suez Canal University Hospital Blood Bank. Blood Disord Transfus.

[49] Ethiopia Mini Demographic and Health Survey 2014. DHS 2011 data analyzed and reported in: HIV/AIDS in Ethiopia. An epidemiological synthesis. Federal in HIV/AIDS Prevention and Control Office 2014. Available at https://www.unicef.org/ethiopia/Mini_DHS_2014__Final_Report.pdf. Accessed 7 Jan 2018.

[50] Apata IW, Averhoff F, Pitman J, Bjork A, Yu J (2014). Progress toward prevention of transfusion-transmitted hepatitis B and hepatitis C infection-sub-Saharan Africa, 2000-2011. MMWR Morb Mortal Wkly Rep.

[51] Abebe A, Nokes DJ, Dejene A, Enquselassie F, Messele T, Cutts FT (2003). Seroepidemiology of hepatitis B virus in Addis Ababa, Ethiopia: transmission patterns and vaccine control. Epidemiol Infect.

[52] Erena AN, Tefera TB (2014). Prevalence of hepatitis B surface antigen (HBsAg) and its risk factors among individuals visiting Goba general hospital, south East Ethiopia, 2012. BMC Res Notes.

[53] Gelaw B, Mengistu Y (2007). The prevalence of HBV, HCV and malaria parasites among blood donors in Amhara and Tigray regional states. Ethiop J Health Dev.

[54] Anagaw B, Shiferaw Y, Anagaw B, Belyhun Y, Erku W, Biadgelegn F (2012). Seroprevalence of hepatitis B and C viruses among medical waste handlers at Gondar town health institutions, Northwest Ethiopia. BMC Res Notes.

[55] Asfaw NE, Tomas BT (2014). Prevalence of hepatitis B surface antigen (HBsAg) and its risk factors among individuals visiting Goba general hospital, south East Ethiopia, 2012. BMC Res Notes.

[56] McCarthy MC, El-Tigani A, Khalid IO, Hyams KC (1994). Hepatitis B and C in Juba, southern Sudan: results of a serosurvey. Trans R Soc Trop Med Hyg.

[57] Elfaki AMH, Eldour AAA, Elsheikh NMH (2008). Sero-prevalence of immunodeficiency virus, hepatitis B and C and syphilis amongblood donors at ElObeid Teaching Hospital, West Sudan. Sudan JMS.

[58] Emmanuel CO, John CA, Theodora UE, Nancy CI, Gloria AN, Israel OO, Christian EO (2015). The epidemiology of transfusion-transmissible infections among blood donors in Nnewi, south-East Nigeria. Afr J Med Health Sci.

[59] Kassim OD, Oyekale TO, Aneke JC, Durosinmi MA (2012). Prevalence of seropositive blood donors for hepatitis B, C and HIV viruses at the Federal Medical Centre, Ido-Ekiti Nigeria. Ann Trop Pathol.

[60] Nagi AM, Altyeb HA, Ahmed AM (2007). Seroprevalence of hepatitis B and C viral infections among blood donors in Shendi, River Nile state, Sudan. Res J Med Medical Sci.

[61] Elsheikh RM, Daak AA, Elsheikh MA, Karsany MS, Adam I (2007). Hepatitis B virus and hepatitis C virus in pregnant Sudanese women. Virol J.

[62] Lauer GM, Walker BD (2001). Hepatitis C virus infection. N Engl J Med.

[63] Taye S, Abdulkerim A, Hussen M (2014). Prevalence of hepatitis B and C virus infections among patients with chronic hepatitis at Bereka medical center, Southeast Ethiopia: a retrospective study. BMC Res Notes.

[64] Ataklti HA, Tsehaye AD, Rashmi B (2016). Sero-prevalence and associated risk factors for hepatitis C virus infection among voluntary counseling testing and anti retroviral treatment clinic attendants in Adwa hospital, northern Ethiopia. BMC Res Notes.

[65] Elsharif AB, Moataz MA, Hamza BH, Omer SM, Mutasim SM, Bader EH (2015). Seroprevalence of viral transfusion-transmissible infections among blood donors at Kosti teaching hospital, White Nile state/Sudan. Int J Curr Microbiol App Sci.

[66] Noubiap JJ, Joko WY, JNansseu JR, Tene UG, Siaka C (2013). Sero-epidemiology of human immunodeficiency virus, hepatitis B and C viruses, and syphilis infections among first-time blood donors in Ed’ea, Cameroon. Int J Infect Dis.

[67] Yusuf M, Alemayehu B (2016). Seroprevalence of transfusion transmitted infection among blood donors at Jijiga blood bank, eastern Ethiopia: retrospective 4 years study. BMC Res Notes.

[68] Martina NA, Okorie OG, Ejike OA (2015). Seroprevalence of human immunodeficiency virus (HIV), hepatitis B surface antigen (HBsAg) and hepatitis C virus (HCV) among voluntary blood donors in Enugu Metropolis. Int J Med Medic Sci.

[69] Ji Z, Li Y, Lv YG (2013). The prevalence and trends of transfusion-transmissible infectious pathogens among first time, voluntary blood donors in Xi’an, China between 1999 and 2009. Int J Infect Dis.

[70] Zahariadis G, Plitt S, O’Brien S, Yi Q, Fan W, Preiksaitis JK (2007). Prevalence and estimated incidence of blood borne viral pathogen infection in organ and tissue donors from northern Alberta. Am J Transplant.

[71] Raina S, Raina S, Kaul R, Sharma V (2015). Seroprevalence of hepatitis B, hepatitis C, human immunodeficiency virus surface, and syphilis among blood donors: a 6-year report from a sentinel site in Western Himalayas, India. Indian J Sexually Transmitt Dis.

[72] Bhawna S, Satish K, Butola K, Mishra J, Yogesh K (2014). Seroprevalence pattern among blood donors in a tertiary health care center. Internet J Medic Update.

[73] Shrivastav A, Bhavsar U, Ramanuj A, Joshi J, Agnihotri A, Bodarya O (2015). Seronegativity HBsAg, HCV and HIV among blood donors: a five year study. Muller J Medic Sci Res.

[74] Bommanahalli B, Javali R, Mallikarjuna CM, Swamy M, Gouda K, Siddartha K, Shashikala K (2014). Seroprevalence of hepatitis B and hepatitis C viral infections among blood donors of Central Karnataka, India. Int J Medical Sci Public Health.

